# Two‐dimensional solid‐state array detectors: A technique for *in vivo* dose verification in a variable effective area

**DOI:** 10.1002/acm2.12744

**Published:** 2019-10-14

**Authors:** Kananan Utitsarn, Giordano Biasi, Nauljun Stansook, Ziyad A. Alrowaili, Marco Petasecca, Martin Carolan, Vladimir L. Perevertaylo, Wolfgang A. Tomé, Tomas Kron, Michael L. F. Lerch, Anatoly B. Rosenfeld

**Affiliations:** ^1^ Centre for Medical Radiation Physics (CMRP) University of Wollongong Wollongong NSW Australia; ^2^ Department of Medical Services Lopburi Cancer Hospital Lopburi Thailand; ^3^ Department of Radiology Faculty of Medicine Mahidol University Bangkok Thailand; ^4^ Physics Department College of Science Jouf University Sakaka Saudi Arabia; ^5^ Illawarra Cancer Care Centre (ICCC) Wollongong Hospital Wollongong NSW Australia; ^6^ SPA‐BIT Kiev Ukraine; ^7^ Department of Radiation Oncology Albert Einstein College of Medicine New York City NY USA; ^8^ Department of Physical Sciences Peter MacCallum Cancer Centre Melbourne Vic. Australia; ^9^ Sir Peter MacCallum Cancer Institute University of Melbourne Melbourne Vic. Australia

**Keywords:** 2D solid‐state array detector, MP512, transmission detector, *in vivo* QA

## Abstract

**Purpose:**

We introduce a technique that employs a 2D detector in transmission mode (TM) to verify dose maps at a depth of d_max_ in Solid Water. TM measurements, when taken at a different surface‐to‐detector distance (SDD), allow for the area at d_max_ (in which the dose map is calculated) to be adjusted.

**Methods:**

We considered the detector prototype “MP512” (an array of 512 diode‐sensitive volumes, 2 mm spatial resolution). Measurements in transmission mode were taken at SDDs in the range from 0.3 to 24 cm. Dose mode (DM) measurements were made at d_max_ in Solid Water. We considered radiation fields in the range from 2 × 2 cm^2^ to 10 × 10 cm^2^, produced by 6 MV flattened photon beams; we derived a relationship between DM and TM measurements as a function of SDD and field size. The relationship was used to calculate, from TM measurements at 4 and 24 cm SDD, dose maps at d_max_ in fields of 1 × 1 cm^2^ and 4 × 4 cm^2^, and in IMRT fields. Calculations were cross‐checked (gamma analysis) with the treatment planning system and with measurements (MP512, films, ionization chamber).

**Results:**

In the square fields, calculations agreed with measurements to within ±2.36%. In the IMRT fields, using acceptance criteria of 3%/3 mm, 2%/2 mm, 1%/1 mm, calculations had respective gamma passing rates greater than 96.89%, 90.50%, 62.20% (for a 4 cm SSD); and greater than 97.22%, 93.80%, 59.00% (for a 24 cm SSD). Lower rates (1%/1 mm criterion) can be explained by submillimeter misalignments, dose averaging in calculations, noise artifacts in film dosimetry.

**Conclusions:**

It is possible to perform TM measurements at the SSD which produces the best fit between the area at d_max_ in which the dose map is calculated and the size of the monitored target.

## 1 INTRODUCTION

Conformal radiotherapy techniques such as intensity‐modulated radiotherapy (IMRT) and volumetric‐modulated arc radiotherapy (VMAT)^1^, ^2^ require accurate verification of treatment plans. Pretreatment quality assurance (QA)^3^ considers point‐dose measurements performed with an ionization chamber^4^ and dose distribution measurements performed with an electronic portal imaging device (EPID),^5^, ^6^, ^7^ a phantom‐based electronic array^8^, ^9^, ^10^, ^11^ or films. However, time‐consuming pretreatment QA is typically considered only once before the first treatment session; potential changes or errors in all sessions will remain unaddressed and/or undetected.^12^, ^13^

An *in vivo* verification approach validates, in real time, accuracy, and integrity of treatment plans; parameters monitored include, for instance, the output of a medical linear accelerator (linac) and the position and/or movement of the leaves of a multileaf collimator (MLC).^12^, ^13^, ^14^, ^15^ Solutions for *in vivo* monitoring include^16^ the use of transit and transmission detectors.

Transit detectors such as EPIDs are placed so that the beam penetrates the patient first, and then the detector.^17^, ^18^, ^19^ QA with transit EPIDs is challenging; their response is energy dependent and there is additional scatter from the patient; also, they are not able to discriminate between changes in signal due to changes in fluence incident on the patient from changes in signal due to anatomical variations within the patient.^20^

Transmission detectors are, instead, placed between the linac head and the patient. Commercially available options include the Device for Advanced Verification of IMRT Delivery (DAVID) system (PTW, Freiburg, Germany), a flat, multiwire transmission‐type ionization chamber^21^, ^22^; the Dolphin detector with the COMPASS software (IBA Dosimetry, Germany), which uses 1513 air‐vented plane parallel ionization chambers,^23^, ^24^, ^25^ the integral quality monitoring (IQM) system (iRT Systems GmbH, Koblenz, Germany), a large‐area wedge ionization chamber^12^, ^13^, ^26^, ^27^; the Delta^4^ Discover (ScandiDos AB, Uppsala, Sweden), a 2D solid‐state array.^16^ Several prototypes have also been proposed in the literature, including optical attenuation‐based scintillating fibers^28^; 2D solid‐state arrays, such as the MP121^29^, ^30^ and the MP512.^31^

Transmission detectors allow for independent monitoring of the output of a linac, and of the position and/or movement of the leaves of an MLC.^16^ However, they have limitations. Any device placed in the beam path affects beam quality and introduces beam attenuation,^12^ and as such has to be modeled in the treatment planning system (TPS).^27^ Also, transmission detectors may increase surface dose^16^, ^23^ and their efficacy for beam monitoring is limited by their shape, active area, and spatial resolution.

The present study introduces a novel technique for using a 2D solid‐state array prototype, the MP512 (512 diode‐sensitive volumes, 2 mm spatial resolution). The MP512 was used in transmission mode (TM) to verify dose maps at a depth of d_max_ in Solid Water. TM measurements were taken at different surface‐to‐detector distances (SDDs) in order to adjust the area at d_max_ where the dose map is calculated.

## 2 MATERIALS AND METHODS

### 2.1 Linear accelerator and treatment planning system

All measurements were performed at the Illawarra Cancer Care Centre (Wollongong, NSW, Australia) using a Varian Clinac^®^ iX (Varian Medical Systems, Palo Alto, CA, USA) linac equipped with a Millennium 120‐MLC with leaf width at the center of 5 mm. The linac operated with a pulse frequency of 360 Hz and was calibrated to deliver 1 cGy/MU at d_max_ in water, at 100 cm source‐to‐surface distance (SSD). In all cases, a 6 MV flattened photon beam was used.

For all dose calculation with a TPS, and for all IMRT plans, we used the Pinnacle's adaptive convolution‐superposition (CS) algorithm implemented into the Pinnacle^3^ TPS version 14 (Philips Medical Systems, Eindhoven, the Netherlands). Dose calculations were performed with a grid of 2 mm. Also, clinical IMRT plans were created, within the TPS, based on computed tomography (CT) datasets of the Solid Water phantom; a SOMATOM CT Scanner (Siemens Healthineers, Erlangen, Germany), acquiring axial slices of 2 mm, was used.

### 2.2 The MP512 system

The MP512 is a prototype of a monolithic silicon‐array detector; it was developed at the Centre for Medical Radiation Physics (University of Wollongong, NSW, Australia). The prototype has 512 diode‐sensitive volumes; these have an area of 0.5 × 0.5 mm^2^ and are uniformly distributed with a pitch of 2 mm over an active area of 52 × 52 mm^2^. The MP512 is operated in passive mode (i.e., no external bias is applied); its associated readout electronics has a high temporal resolution (pulse‐by‐pulse signal acquisition).^32^

In the literature, the MP512 has been characterized as a phantom‐based detector for quality assurance in modern radiotherapy; it was demonstrated to be an accurate dosimeter for the measurement of output factors, percentage depth dose distributions, and lateral‐dose profiles; furthermore, its angular dependence was investigated and corrected for, making it a suitable candidate for quality assurance in arc deliveries.^33^, ^34^, ^35^ The use of the MP512 as a transmission detector was also assessed.^31^ In that study, it was reported that the MP512 in TM increases the surface dose by <25% for a SDD in the range from 0.3 to 18 cm, and by <5% for SDD >18 cm.^31^ The transmission factor, at d_max_ depth in Solid Water, 100 cm SSD, was in the range from 1.020 to 0.997 for SDDs from 0.3 to 24 cm.^31^

### 2.3 Gafchromic™ EBT3 films and Farmer ionization chamber

We considered measurements with Gafchromic™ EBT3 films and with a Farmer NE2571 ionization chamber, performed under the same experimental conditions.

Films were scanned with an EPSON Expression 10000 XL flatbed scanner using a 48‐bit RGB and a resolution of 72 dpi. Films were pre‐ and post‐scanned (24 hrs after irradiation) six times maintaining a consistent orientation and using only the last three optical density maps. Films were calibrated using absolute dose measurements with the Farmer chamber.^36^ Film analysis methodology was the same as that used by Aldosari et al.^37^

### 2.4 Measurements in transmission mode and in dose mode

The MP512's active area was made light‐tight using a black plastic sheet of thickness 80 µm. An equalization procedure, performed prior to all measurement, was used to address a nonuniformity in the integral response of the MP512's sensitive volumes.^38^ Also, to convert readings to absolute dose, the MP512 was calibrated using measurements of response linearity with dose; those measurements were performed in jaws‐defined fields of 10 × 10 cm^2^, at a depth of d_max_ in Solid Water, 100 cm SSD. Delivered MUs were in the range from 1 to 1000 MU, at a fixed dose rate of 600 MU/min. The Farmer chamber was used for the absolute dose measurements at a depth of d_max_ in Solid Water.^36^

**Figure 1:**
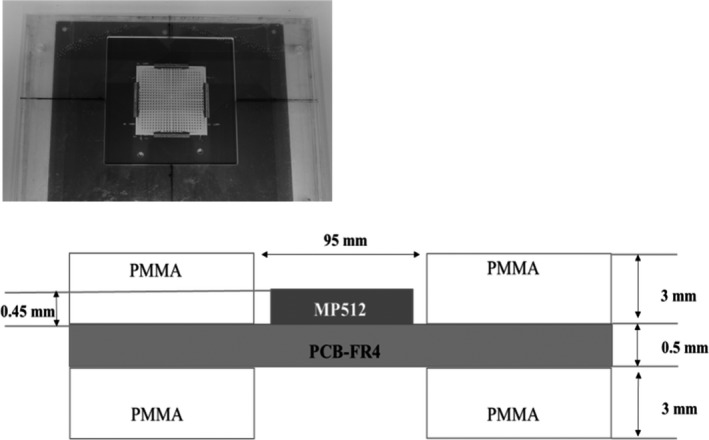
(Upper panel) A snapshot of the active area of the MP512 and protective PMMA slabs. (Lower panel) A schematic of the packaging of the MP512 system, with the MP512 active area proper, upper and lower PMMA protective slabs, and the PCB‐FR4 board on which the detector is wire bounded for signal readout.

**Figure 2:**
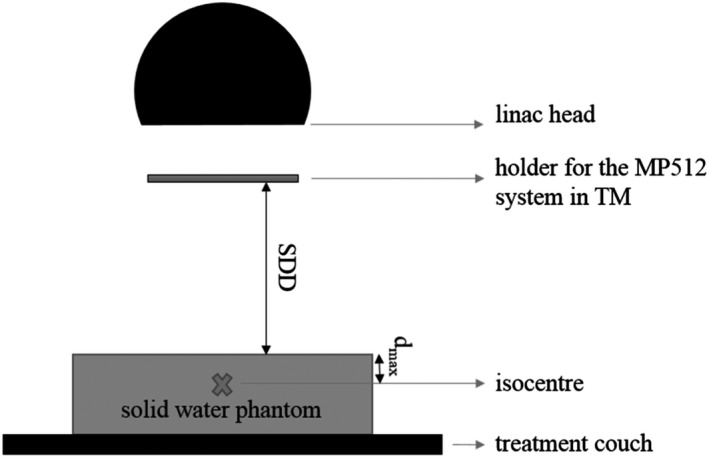
Use of the MP512 system in transmission mode (not to scale).

For TM measurements, the MP512 was sandwiched between two protective slabs of PMMA of thickness 3 mm. To minimize the resulting composite thickness, each slabs had an opening, centered on the axis of the MP512's active area, of 9.5 × 9.5 cm^2^ (Figure 1). The MP512 was then lodged into a movable holder of PMMA; by moving the holder, the SDD could be varied in the range from 0.3 to 24 cm (Figure 2). The effective area (A_eff_), at a depth of d_max_ in Solid Water, was defined as a function of SDD as:

(1) $$A_{\text{eff}}=A_{\text{MP}512}{\left(\frac{\text{SSD}+1.5}{\text{SSD}-\text{SDD}}\right)}^{2},$$

with A_MP512_ the MP512's active area.

For dose mode (DM) measurements, the MP512 was placed at a depth of d_max_, in Solid Water on the treatment couch. In that case, the MP512 was sandwiched between two slabs of PMMA of thickness 5 mm; the top slab had a small recess (or air gap),^39^ centered on the axis of the MP512's active area, of thickness 0.5 mm. The air gap was necessary to minimize, in small radiation fields,^40^ the number and size of corrections required to relate the MP512's readings to dose.^41^

TM and DM measurements were performed in jaws‐defined static fields of 2 × 2 cm^2^, 3 × 3 cm^2^, 5 × 5 cm^2^, 8 × 8 cm^2^, and 10 × 10 cm^2^, as defined at 100 cm SSD, delivering 200 MU at 600 MU/min. All measurements were repeated three times to minimize random uncertainties and errors were calculated as one standard deviation. In all fields, the ratio between DM measurements and TM measurements, as a function of SDD, was fit using the least square method.

### 2.5 Dose calculations in static square fields and IMRT fields

The response of the MP512 in TM was measured in static fields of 1 × 1 cm^2^ and 4 × 4 cm^2^, at 4 and 24 cm SDD. In each of these fields, the response of the MP512 in DM at d_max_ was then calculated using the relationship between DM and TM measurements derived as described in the previous section. Note that these fields were not part of those used to obtain the relationship in the first place. As the field of 1 × 1 cm^2^ was smaller than the smallest field used for the fit, the calculated response in DM was extrapolated. The response in DM in the square field of 4 × 4 cm^2^ was calculated by interpolation. Calculated responses in DM were then compared with responses in DM measured with the MP512 itself, with Gafchromic™ EBT3 films and with a Farmer ionization chamber.

Additionally, the response of the MP512 in TM was measured at 4 and 24 cm SDD in clinical IMRT fields; these fields were delivered with a treatment plan used to treat a malignant base of skull chordoma. The step‐and‐shoot plan, consisting of six static fields defined by the MLC, delivered a nominal dose 1.8 Gy per fraction to a gross tumor volume (GTV) of 12.40 cm^3^. All fields were delivered with the gantry at 0° (incident beam perpendicular to the active area of the MP512) to rule out angular dependence effects on the response.^35^, ^42^ Equivalent square fields (A_eq_) of IMRT fields were calculated using^43^:

(2) $$A_{\text{eq}}=\frac{2\text{xy}}{x+y}$$

As above, in each of these fields, the response of the MP512 in DM at d_max_ was then calculated using the relationship between DM and TM measurements. Calculated dose distributions were compared with TPS calculations and with DM measurements with the MP512 itself and with Gafchromic™ EBT3 films. The comparison was performed with a gamma index analysis with the following acceptance criteria: 1%/1 mm, 2%/2 mm, and 3%/3 mm; a global threshold of 10% was applied.

## 3 RESULTS

### 3.1 Measurements in transmission mode and in dose mode

**Figure 3:**
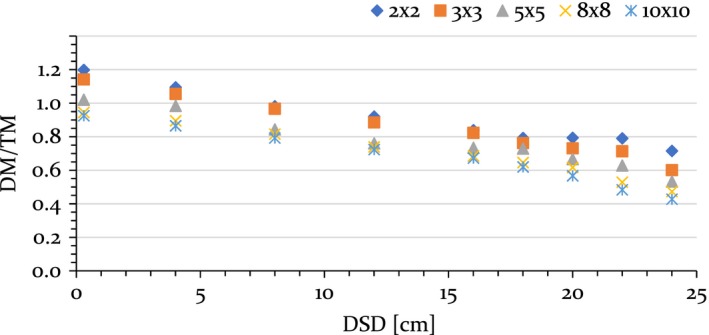
Central sensitive volume of the MP512: ratio of dose mode measurements to transmission mode measurements as a function of surface‐to‐detector distance, for all considered square fields. Error bars did not exceed symbol size.

Figure 3 shows ratios between DM and TM measurements with the central sensitive volume of the MP512. Ratios, a function of field size and SDD, were fit; corresponding slopes (M) and axis intercepts (B_A0_) are in Table 1. It was observed that M depends weakly on field size. Based on this result, it was chosen to work with its value averaged across all considered fields. Also, to compute M and B_A0_, we operated on the central sensitive volume first; we then repeated the procedure for all other sensitive volumes — all volumes had values of M and B_A0_ in agreement to within 1.79%.

**Table 1:** Central sensitive volume of the MP512: slope (M) and axis intercept (B_A0_) of the ratio of dose mode measurements to transmission mode measurements, for all considered square fields; absolute values.

Parameter	Square field, side [cm]				
	2	3	5	8	10
M	0.020	0.021	0.018	0.019	0.020
B_Ao_	1.165	1.144	1.028	0.969	0.953

Using the averaged M (0.0196) and the B_A0_ value corresponding to any given field size, the dose in DM at d_max_ was calculated using the TM measurement at a given SDD as:

(3) $$\text{DM}={\text{TM}}_{\text{SDD}}\times \left(B_{A0}-M\times \text{SDD}\right)$$

**Figure 4:**
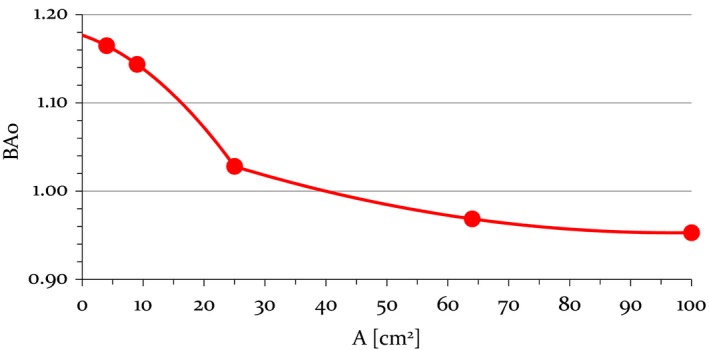
Ratio of measurements in dose mode at d_max_ to measurements in transmission mode: B_Ao_ as a function of radiation field area A. Error bars did not exceed symbol size.

For an arbitrary radiation field of area A, B_A0_ could be found from the piecewise polynomial fit (adjusted regression coefficient R^2^ = 1) (Figure 4):

(4) $$B_{Ao}=-0.000142\times A^{2}-0.002392\times A+1.176743,$$

for 0 cm^2^ ≤ A ≤ 25 cm^2^, and

(5) $$B_{Ao}=0.000015\times A^{2}-0.002822\times A+1.089544,$$

for 25 cm^2^ ≤ A ≤ 100 cm^2^.

### 3.2 Dose calculations in regular static fields and IMRT fields

B_A0_ values relative to static fields of 1 × 1 cm^2^ and 4 × 4 cm^2^ side, and to IMRT fields, were calculated using Equations 4 and 5.

Table 2 shows calculated dose at d_max_ (using TM measurements at 4 and 24 cm SDD) along with corresponding TPS calculations and DM measurements performed with the MP512 itself, with Gafchromic™ EBT3 films, and with a Farmer ionization chamber.

**Table 2:** Static square field of 1 and 4 cm side: calculated dose [Gy] at d_max_ (using transmission mode measurements at 4 and 24 cm SDD) compared with treatment planning system (TPS) calculations and dose mode measurements performed with the MP512, with films and with a Farmer ionization chamber.

Square field, side [cm]	MP512, calc.		MP512, meas.	TPS	EBT3	Farmer chamber
	4 cm SDD	24 cm SDD				
1	0.798	0.821	0.816	0.810	0.809	0.813
4	1.013	1.020	1.004	0.996	0.998	1.010

Table 3 shows gamma passing rates (%GP) between calculated dose distributions for IMRT fields at d_max_ (using TM measurements at 4 and 24 cm SDD) and corresponding TPS calculations and DM measurements performed with the MP512 itself and Gafchromic™ EBT3 films.

**Table 3:** Intensity‐modulated radiotherapy fields: gamma evaluation for dose calculations at d_max_ (using transmission mode measurements at 4 and 24 cm SDD) and corresponding treatment planning system calculations and dose mode measurements performed with the MP512 and with films.

SDD [cm]	Acceptance criteria								
	3%/3 mm			2%/2 mm			1%/1 mm		
	MP512, calc. vs. TPS	MP512, calc. vs. EBT3	MP512, calc. vs. MP512, meas.	MP512, calc. vs. TPS	MP512, calc. vs. EBT3	MP512, calc. vs. MP512, meas.	MP512, calc. vs. TPS	MP512, calc. vs. EBT3	MP512, calc. vs. MP512, meas.
4	98.14%	96.89%	99.79%	90.50%	92.00%	98.59%	62.20%	69.40%	99.40%
24	97.22%	97.53%	99.69%	93.80%	93.80%	97.69%	59.00%	71.00%	99.00%

## 4 DISCUSSION

A relationship was derived (Equation 3) for calculating dose, at a depth of d_max_, by using TM measurements with the MP512 at a given SDD, in any given field. The relationship was used to calculate dose at d_max_ by using TM measurements, at 4 and 24 cm SSD, in static fields of 1 × 1 cm^2^ and 4 × 4 cm^2^. Calculations agreed to within ±2.36% (mean difference 1.43%) with TPS calculations and DM measurements performed with the MP512 itself, with Gafchromic™ EBT3 films, and with a Farmer ionization chamber.

The relationship was also used to calculate dose at d_max_ by using TM measurements, at 4–24 cm SSD, in step‐and‐shoot clinical IMRT fields. Calculated dose maps had %GP, when compared with TPS calculations and film dosimetry, greater than 96.89%, 90.50%, 62.20% (SDD 4 cm) and greater than 97.22%, 93.80%, 59.00% (SDD 24 cm), using acceptance criteria of 3%/3 mm, 2%/2 mm, and 1%/1 mm, respectively.

In the clinical practice, dose distributions are typically compared using gamma index analysis,^44^, ^45^, ^46^ with a clinically significant acceptance criterion of a 3% dose difference (%DD) and 3 mm distance‐to‐agreement (DTA).^1^, ^47^, ^48^ In the present study, more stringent criteria were also considered for completeness. Our dose calculations had significantly lower %GP when considering a 1%/1 mm acceptance criterion. This result can be explained by factors such as submillimeter misalignments in TM detector positioning, dose averaging in TPS calculations over a 2 mm grid, noise artifacts created by film heterogeneities, as well as handling and scanning procedures. Misleading results from the gamma index analysis may also originate from the use of detectors with a resolution not appropriate for the selected acceptance criterion.^49^ The use of a 2D solid‐state detector prototype in TM with a higher resolution than the MP512, such as the Octa (0.3 mm),^50^, ^51^ would help to shed light.

Note that, in the present study, the MP512 was not modeled into the TPS. Its transmission factor was reported to be in the range from 1.020 to 0.997 for SDDs from 0.3 to 24 cm.^31^ However, if clinical use will be considered, it is suggested that transmission factor has to be adequately incorporated into a TPS.^12^

The effective area, at a depth of d_max_ in Solid Water (Equation 1), in which dose maps could be calculated based on TM measurements varied in the range from 28 cm^2^ (SDD 0.3 cm) to 48.2 cm^2^ (SDD 24 cm); those values reflected a MP512 having an active area of 27.04 cm^2^. Depending on the clinical application, a 2D detector of larger active area may be required.

Using the MP512 in TM lodged on a holder positioned away from the linac head has the additional advantage of minimizing the contribution of scattered electrons, so that the detector response is mostly driven by the photon energy fluence, potentially simplifying 3D dose reconstructions at d_max_ in phantom.

Our study, a preliminary investigation, had the limitations of considering only 6 MV flattened photon beams, of not assessing the DM to TM ratio in fields off‐axis, and of using a unique B_A0_ value for both jaws‐defined and MLC‐defined fields, also neglecting the influence of the backup jaws.

## 5 CONCLUSION

The introduced technique uses a variable SDD for transmission mode (TM) measurements with a 2D detector. In this way, dose maps at a depth of d_max_ in Solid Water are calculated in an effective area (A_eff_) tailored to the size of the monitored target.

When considering a gamma index analysis with a strict 1%/1 mm acceptance criterion, lower gamma passing rates (%GP) between our dose calculations and benchmarks (treatment planning system calculations, film dosimetry), which can be due to submillimeter misalignments in detector positioning or dose averaging in calculations, emphasize the importance of developing array detectors with high‐spatial resolution.

This study represents a first step in the development of a real‐time high‐resolution 3D dose reconstruction technique based on TM measurements with the MP512 prototype.

## CONFLICTS OF INTEREST

The authors declare they have no conflict of interest.
